# Exploring the merits of research performance measures that comply with the San Francisco Declaration on Research Assessment and strategies to overcome barriers of adoption: qualitative interviews with administrators and researchers

**DOI:** 10.1186/s12961-023-01001-w

**Published:** 2023-06-05

**Authors:** Himani Boury, Mathieu Albert, Robert H. C. Chen, James C. L. Chow, Ralph DaCosta, Michael M. Hoffman, Behrang Keshavarz, Pia Kontos, Mary Pat McAndrews, Stephanie Protze, Anna R. Gagliardi

**Affiliations:** 1 Toronto General Hospital Research Institute, University Health Network, Toronto, Canada; 2 The Institute for Education Research, University Health Network, Toronto, Canada; 3 Research Solutions and Services, University Health Network, Toronto, Canada; 4 Techna Institute, University Health Network, Toronto, Canada; 5 Princess Margaret Cancer Centre, University Health Network, Toronto, Canada; 6 KITE Research Institute, Toronto Rehabilitation Institute, University Health Network, Toronto, Canada; 7 Krembil Research Institute, University Health Network, Toronto, Canada; 8 McEwen Stem Cell Institute, University Health Network, Toronto, Canada

**Keywords:** Research personnel, Performance appraisal, Academic review, Indicators, Criteria, Acceptance, Barriers, Implementation, Qualitative interviews, San Francisco Declaration on Research Assessment

## Abstract

**Background:**

In prior research, we identified and prioritized ten measures to assess research performance that comply with the San Francisco Declaration on Research Assessment, a principle adopted worldwide that discourages metrics-based assessment. Given the shift away from assessment based on Journal Impact Factor, we explored potential barriers to implementing and adopting the prioritized measures.

**Methods:**

We identified administrators and researchers across six research institutes, conducted telephone interviews with consenting participants, and used qualitative description and inductive content analysis to derive themes.

**Results:**

We interviewed 18 participants: 6 administrators (research institute business managers and directors) and 12 researchers (7 on appointment committees) who varied by career stage (2 early, 5 mid, 5 late). Participants appreciated that the measures were similar to those currently in use, comprehensive, relevant across disciplines, and generated using a rigorous process. They also said the reporting template was easy to understand and use. In contrast, a few administrators thought the measures were not relevant across disciplines. A few participants said it would be time-consuming and difficult to prepare narratives when reporting the measures, and several thought that it would be difficult to objectively evaluate researchers from a different discipline without considerable effort to read their work. Strategies viewed as necessary to overcome barriers and support implementation of the measures included high-level endorsement of the measures, an official launch accompanied by a multi-pronged communication strategy, training for both researchers and evaluators, administrative support or automated reporting for researchers, guidance for evaluators, and sharing of approaches across research institutes.

**Conclusions:**

While participants identified many strengths of the measures, they also identified a few limitations and offered corresponding strategies to address the barriers that we will apply at our organization. Ongoing work is needed to develop a framework to help evaluators translate the measures into an overall assessment. Given little prior research that identified research assessment measures and strategies to support adoption of those measures, this research may be of interest to other organizations that assess the quality and impact of research.

**Supplementary Information:**

The online version contains supplementary material available at 10.1186/s12961-023-01001-w.

## Background

The San Francisco Declaration on Research Assessment (DORA) was established in 2012 during the Annual Meeting of the American Society for Cell Biology [1]. DORA principles advocate for research assessment based on a broad range of discipline-relevant measures of quality and impact, and eliminating journal-based metrics such as Journal Impact Factor. DORA recommends that academic organizations be explicit about criteria used for hiring, annual review, tenure, and promotion decisions; assess the value and impact of all research outputs in addition to research publications; and consider a broad range of measures including qualitative indicators of research impact such as influence on policy and practice. As of 1 November 2022, 22,311 individuals and organizations in 159 countries are DORA signatories.

Some groups have generated principles for assessing research that align with the DORA statement. For example, the Leiden Manifesto includes ten principles such as measure performance against research institute mission, and accounts for variation by field in publication and citation practices [2]. A 2017 meeting of international experts in scientific communication generated five principles upon which to judge research: assess contribution to societal needs, employ responsible indicators, reward publishing of all research regardless of the results, recognize the culture of open research, fund research that generates evidence on optimal ways to assess science and faculty, and fund/recognize out-of-the-box ideas [3]. While helpful, these principles offer high-level guidance but not concrete performance measures. Others have suggested measures for research assessment, but they are discipline-specific and not broadly applicable to diverse fields of research. For example, Mazumdar et al. proposed criteria to assess the contributions of biostatisticians to team science [4].

To address these limitations, our group identified and prioritized measures of research quality and impact. To be clear, we included only measures of research activity and outputs, and excluded measures pertaining to teaching, mentoring, and other service. The methods and results are reported elsewhere [5]. In brief, we synthesized peer-reviewed research and grey literature, including documents from Canadian academic organizations and international scholarly organizations that had adopted DORA principles, to generate a list of 50 unique measures for assessing the quality and impact of research. We then conducted a two-round Delphi survey of multidisciplinary researchers, research administrators, and research leaders to achieve consensus on priority measures. This resulted in ten measures organized in eight domains: relevance of research program, challenges to research program or productivity, team/open science, funding, innovations, publications, other dissemination, and impact. The measures can be used by researchers across disciplines in our organization and beyond to describe their research achievements, and by various staff in academic organizations to support hiring, annual review, tenure, promotion, and other decisions based on the quality and impact of research.

While the limitations and harms of evaluating research on the basis of metrics such as Journal Impact Factor have long been recognized [6–11], research assessment has largely focused on journal metrics for quite some time. For example, a 2018 survey of criteria used for assessing researchers at 92 international faculties of biomedical sciences revealed they largely employed traditional measures such as the number of peer-reviewed publications, impact factor, and number or amount of grant funding [12]. Thus, it may be challenging to promote adoption of measures that do not include journal metrics, and instead rely on assessing the quality and impact of the research itself rather than where it was published. The overall aim of this study was to explore how to promote acceptance and use of the ten priority measures. The specific objectives were to identify perceived strengths and limitations of the measures, and suggestions for strategies needed to overcome barriers and support adoption of the measures. Such knowledge is critical for planning implementation of the measures including approaches, interventions, or tools.

## Methods

### Approach

We employed a qualitative research design to fully explore participant perspectives on the ten priority measures [13]. More specifically, we used qualitative description, an approach that does not test or generate theory [14]. Instead, this approach is commonly used in health services research to gather explicit information about views, experiences, and suggestions. We complied with the Consolidated Criteria for Reporting Qualitative Research checklist [15]. The University Health Network Research Ethics Board granted ethical approval for this study (REB #22–5082). All participants provided written informed consent prior to interviews. The researchers had no relationship with the participants, other than employment at the same institution. The University Health Network is one of Canada’s largest academic hospitals offering 12 medical programs across 10 hospital sites. The research arm of the University Health Network is organized into distinct research institutes that vary in size, administration, and focus including cardiology, transplantation, neurosciences, oncology, surgical innovation, infectious diseases, genomic medicine, healthcare education, and rehabilitation medicine.

### Sampling and recruitment

We used purposive sampling to recruit individuals from our organization whose views about the measures might vary by role (researcher, research administrator, research leader) and research discipline (six research institutes spanning biomedical, clinical, health services, population health, rehabilitation, and medical education research). We identified eligible persons on publicly available research institute web sites, and also used snowball sampling by first interviewing research administrators in different research institutes and asking them to refer us to others in their research institute. We aimed to recruit a single administrator (e.g., business manager) and leader (e.g., scientific director) plus one researcher from each research institute, aiming to also include in non-mutually exclusive fashion persons representing early, mid-, and later career, for a target of 18 interviews. In qualitative research, sampling is concurrent with data collection and analysis to the point of thematic saturation, or when no further unique themes emerge from successive interviews, which was established through discussion of themes by the research team. This is consistent with the 12–15 interviews by when saturation is often achieved [16]. We first contacted potential participants by email on 28 April 2022, followed by email reminders to non-respondents every two weeks until we closed recruitment on 3 August 2022. Following informed consent, we used email to schedule the interview and share the ten priority measures (Additional File 1), asking participants to review the measures in advance and have the measures available during the interview.

### Data collection

We conducted a single telephone interview with participants through May and July 2022. A.R.G. (PhD-trained woman Senior Scientist/Professor with extensive qualitative experience) conducted the first four interviews while H.B. (MPH-trained woman Research Associate with some qualitative experience) attended for training purposes, and the next two were conducted by H.B. while A.R.G. attended, followed by discussion to further support training. Thereafter, H.B. conducted all interviews, with periodic review by A.R.G. to provide feedback and answer questions posed by H.B. Interview questions were reviewed and refined by the research team prior to use. The interview guide (Additional file 2) included five questions related to perceived strengths of the measures, perceived limitations of or gaps in the measures, potential barriers to reporting the measures, potential barriers to assessing the measures, and strategies needed to facilitate use of the measures for reporting or assessing research. The interview guide included prompts that we invoked only if any participant provided little response to a given question. Prompts were informed by what we heard from participants of early interviews. Both questions and prompts were posed in an open, non-leading manner to avoid influencing responses. Interviews ranged from 15 to 60 min, and were audio-recorded and transcribed.

### Data analysis

We used content analysis to identify themes inductively through constant comparison and used Microsoft Office (Word, Excel) to manage data [13, 14]. H.B. and A.R.G. independently coded the first three interviews, then compared and discussed coding to develop a preliminary codebook of themes and exemplar quotes (first-level coding). H.B. coded subsequent interviews to expand or merge themes (second level of coding). H.B. met with A.R.G. periodically to discuss and refine coding. We tabulated data (themes, quotes) by participant institute, participant role, participant career stage, and research discipline to compare themes. We used summary statistics to describe participants and text to describe key themes. As is customary in qualitative research, we used words like few or many to convey whether some or most of the participants articulated a given idea as a way of exploring and reporting major discrepancies in the views or suggestions of participants.

## Results

### Participants

We interviewed a total of 18 participants: 6 administrators (research institute business managers and directors) and 12 researchers (7 of whom were on research institute appointment committees). Participants varied by research institute, thus representing the perspectives of various research disciplines (Table 1). The career stage of the 12 researchers and directors included 2 early, 5 mid-, and 5 later career stage.

**Table 1:** Participant characteristics

Research institute	Role		Subtotal
	Researcher	Administrator	
1. Rehabilitation medicine	2 (1 appointment committee)	1	3
2. Brain diseases	0	1	1
3. Stem cell research	1	1	2
4. Oncology	3 (2 appointment committee)	2	5
5. Healthcare education	2 (2 appointment committee)	1	3
6. Biomedical/health services	2 (1 appointment committee)	2	4
Subtotal	10	8	18

### Themes

Data (themes and quotes) are available in Additional file 3. Table 2 lists themes with exemplar quotes. Here we discuss themes organized by interview question with select illustrative quotes, and highlight any views that differed among participants.

**Table 2:** Themes and exemplar quotes

Theme	Exemplar quote
Perceived strengths of the measures	
Measures cover the major aspects of being a productive researcher/comprehensive	Using these ten criteria as an assessor, I think I can get a pretty good feel for what that researcher is doing and what their successes have been and what their challenges have been (012 JS mid clinical)
Measures are relevant and applicable to different research disciplines	I think one of the strengths is the breadth of and the various different angles that are being used to assess the strengths of a research program that takes into consideration or acknowledges the fact that there is a wide range of research and research approaches being conducted whether it is at UHN or at another organization. (017 BM)
Moving away from impact factor allows researchers to personalize reporting, resulting in fair assessment	What I perceive as the strengths of the measures is the fair assessment of research advances and of individuals involved in research. (05 AC late biomedical)
Measures are similar to those in current use	These are all the same measures that we at < institute > use for our evaluations. It’s just structured in a nice way (01 AC late biomedical)
Easy to understand and use; response options are limited to key achievements, which minimizes reporting burden	It’s fairly clear and reasonably concise. Everybody that’s filling out this kind of form is busy…Its nice to have something that’s a straightforward template. (014 AC late biomedical)
Measures were generated using a rigorous scientific process	And I was thinking that a lot of it is very science-based and makes sense for UHN. (02 AC early health services)
Referring to specific measures:	*Challenges to productivity* Where it talks about describing challenges based in the last 5-years, I think is a good way of accounting for things that might have changed, pregnancy or pandemics or changes in position or those kinds of things. (06 AC mid biomedical)
	*Collaboration* I don’t think we explicitly ask them about their collaborative work. We infer this information from these group grants. The question here is a little cleaner in a sense that it gets them to describe it and to explain how they’re advancing knowledge or having a larger impact through their collaborative work. It’s a little bit more explicit than what we did before. (018 BM)
	*Research contributions* I like the list of key research outputs. It gives you things like databases and computational and informatics tools and public domain resources that are so important these days. Researchers might not always be published in a traditional peer-review paper. I really like that you have it explicitly stated those kinds of outputs are something that can be highlighted (01 AC late biomedical)
Perceived limitations or gaps in the measures	
Measures do not reflect non-research activities such as teaching or service	There’s a lot more that goes into what we do, and I understand that this is supposed to be focused on research, but many of us are also teachers, many of us are mentors and this sort of moves into the realm of not acknowledging those contributions which are fundamental to the operation of UHN Research that really aren’t captured by this. And I think it takes an awfully narrow scope of what’s involved in our roles here. (013 S early biomedical)
Output options listed are not comprehensive:	*Medical education research* Adding curriculum onto that would capture the health professions education element more appropriately (02 AC early health services) *Intangible outputs of community-based research collaboration* We are going to be engaging in co-creation of some kind of a written project, possibly a paper, if that’s what suits their community. Fitting in some of the indigenous requirements for research like ownership of data and that their research belongs to the community and goes back to the community, and doesn’t necessarily turn into a publication (02 AC early health services)
Measures do not include the effort of failed attempts to capture research funding	There should be some attempt to glean some kind of learning from failed attempts as well, especially on large grants, on what did they learn out of the entire process of applying for a big grant and not getting it. Maybe those kinds of things could be collated and made available to other people who are going on to apply for such grants in the future. (010 BM)
Measures may not be relevant across research disciplines	I think the disciplines are so different that to use this collection of measures equally across disciplines is extremely difficult (06 AC mid biomedical)
Five-year time frame is too short and may not reflect impact and quality	One of the deficiencies is that it focuses very much on the last 5 years and sometimes it’s important to put things into context over a longer research career (016 ID late biomedical)
Potential barriers to reporting the measures (researchers)	
Time consuming or requires effort	It’s gonna be a lot of work for people to do this on an annual basis, because as I understand it, the administrative staff goes in, copy from their CV…they assist with pulling these publications into the annual activity report or it’s automatically pulled up by the research analytics group…This one’s gonna take a little bit more work to describe how their research is leading to a certain outcome (018 BM)
Some people are better at describing their research than others but this does not reflect the quality and impact of their research	People differ in their ability to describe the impact of their work but that does not necessarily reflect the impact of the work, it reflects their ability to describe it (09 ID mid biomedical)
Potential barriers to assessing measures (evaluators)	
Inertia or reluctance to implement or adopt the measures	Acceptance of the measures, because any kind of change is difficult. So this is very new way of assessing research and I think it requires a lot of shift in that we deeply hold on to what we’ve been doing, the norms of assessment, and I think the shift to these kinds of measures is the biggest challenge instead of the measures themselves or gaps in the measures (010 BM)
Increased workload to assess merits of publications	There’s an expectation that evaluators will actually read the paper and make an assessment of the value of that research from the research article itself, not just from the title of the journal (01 AC late biomedical)
Unclear how to weight different measures to generate or distinguish an overall evaluation	You can’t just count the number of publications and so on. You have to take into account whether sitting on an international panel of experts to determine a set of guidelines or core outcome measures or something is equivalent to one publication or two publications and so on. (011 SS late clinical)
Without impact factor, may be difficult to assess research in disciplines that are new or unfamiliar, leading to biased or inaccurate assessments	If it’s someone from outside of their field, how are they gonna know whether that’s a significant contribution or not…And I think that that’s the place where bias could be an issue (02 AC early health services)
Strategies needed to address barriers and support adoption of the measures	
Allow research institutes to decide if to apply select measures	I think if a research institute, something didn’t kind of resonate with them or wasn’t as important; I think perhaps they could choose not to use one of those measures (04 BM)
Researchers and evaluators will comply if measures are formally endorsed as the standard and become normalized	First, it comes from leadership. Leadership has to implement this over any formalized protocol or strategy that’s currently used for evaluation of scientists (07 AC mid biomedical)
Hold an official launch and communicate in various ways to raise awareness on why this is important	We do town halls and open forum, that kind of a thing to better socialize it and make it an open discussion and gather people’s comments and their apprehensions and appreciation of the entire process (010 BM)
Ensure that researchers have admin support or automate the reporting process	Is there smart text to link our CV’s in some UHN format and then you can link what they’re looking for in the DORA document to your CV to make it even more efficient. (012 JS mid clinical)
Inform researchers about the metrics so that they can track them	Knowing these in advance, so if I was preparing my career, knowing these in advance, these can all be tracked. (06 AC mid biomedical)
Train researchers or appointment committee members on how to report or assess the measures	Maybe some brief training modules for people who are serving on these various committees could be helpful. (07 AC mid biomedical)
Provide guidance to evaluators on how to assess and interpret measures	We also really need guidance on what is considered a good job and not a good job when we no longer have those numbers to attach ourselves to (017 BM)
Evaluators should be from same discipline as researcher under review	There should be at least one reviewer who assesses that from within that specialty or area of research. (03 AC mid health services and population)
Employ multiple evaluators	If you have more than one person in the assessing these indicators, then hopefully you would have convergence at some point on what the outcome is. (011 SS late clinical)
Evaluators must contextualize measures to both discipline and career stage	You have to put in the context of the person you’re reviewing (08 BM)

*BM* business manager, *AC* appointment committee scientist, *JS* junior scientist, *SS* senior scientist, *ID* institute director

#### Perceived strengths of the measures

Overall, participants identified numerous benefits of the measures and the template for reporting the measures. Most participants said that the measures covered the major aspects of being a productive researcher, many using the word “comprehensive,” and that the measures were equally relevant to different research disciplines. In particular, by moving away from impact factor, the measures would allow researchers to personalize reporting according to their research discipline, resulting in fair assessment. Most researchers and one administrator said that the measures were relatively similar to those already in use, suggesting that little change was needed to adopt the measures. Similarly, most researchers and one administrator said the measures were easy to understand, and the template was easy to use because it provided options to choose from and limited open-ended responses to a few key achievements. One researcher appreciated that the measures were generated using a rigorous scientific process, referring to the literature search, environmental scan and Delphi survey process we used to identify and prioritize the measures [5].

Using these ten criteria as an assessor, I think I can get a pretty good feel for what that researcher is doing and what their successes and challenges have been (012 JS mid clinical).

Several participants noted the merits of specific measures. For example, participants appreciated the opportunity to describe challenges to their productivity “because the real world does impact research.” Others highlighted the novelty of describing collaboration, an important aspect of research yet not typically explicitly reported or assessed in the past. Participants also appreciated multiple opportunities to describe their research contributions including a list of key outputs in addition to publications, how their research directly or indirectly improves health or health care, how their research advances knowledge, and any other relevant information about the quality and impact of their research not captured by other measures.

Researchers might not always be published in a traditional peer-review paper. I really like that you have it explicitly stated those kinds of outputs are something that can be highlighted (01 AC late biomedical).

#### Perceived limitations or gaps in the measures

Compared with strengths, fewer participants raised concerns about the measures. Regarding limitations, the most commonly articulated concern was that the measures may not be relevant across research disciplines. This was mentioned by five administrators, including two who had noted that relevance across disciplines was a strength, and a single researcher. Several participants also said that the 5-year time frame (reflecting the 5-year review for scientists at our institution) was too short given that some years are more productive than others, and in some research disciplines, achievements may only be realized in the context of an entire career.

I think the disciplines are so different that to use this collection of measures equally across disciplines is extremely difficult (06 AC mid biomedical).

In terms of gaps, a few participants said that the measures did not reflect activities such as teaching, mentorship, and other service, which they viewed as a major and important part of what researchers do. A single researcher noted that options listed as examples of research outputs did not include medical curriculum and outcomes associated with community-based research collaboration. A single administrator noted the absence of a measure to capture the effort of failed attempts to capture research funding.

#### Potential barriers to reporting the measures

Few participants identified barriers that researchers may face when reporting the measures. Some thought that it would be time-consuming to prepare descriptive accounts in response to these measures, referring to the fact that, in the past, information about grants and publications was gathered by others from their CVs. The same administrators and other researchers said that some people are better able to describe their research than others, suggesting that poorly written descriptive accounts would not reflect research quality and impact.

People differ in their ability to describe the impact of their work but that does not necessarily reflect the impact of the work, it reflects their ability to describe it (09 ID mid biomedical).

#### Potential barriers to assessing the measures

Compared with barriers of reporting the measures, more participants identified barriers that evaluators may face when assessing the measures. Without being able to rely on journal impact factor, which they viewed as objective, participants said that it would be difficult to judge research outside of one’s own discipline, possibly leading to biased or inaccurate assessments. Furthermore, without numerical data such as Journal Impact Factor, evaluators might be unclear how to weight different measures to generate an overall evaluation, or to distinguish between candidates in the scenario of hiring. Given these concerns, a single researcher said that the workload would be greater because they would have to read publications to assess research contributions rather than relying on Journal Impact Factor. Several administrators and researchers highlighted that many are comfortable with the status quo, and reluctant to accept the changes required to implement or adopt the measures.

The shift to these kinds of measures is the biggest challenge instead of the measures themselves or gaps in the measures (010 BM).

#### Strategies needed to address barriers and support adoption of the measures

Three administrators suggested that research institutes be allowed to choose which of the ten measures to adopt, in contradiction to the many participants who said that the measures are similar to those in current use. This view was qualified by two participants: one administrator said that it would simply take time to adjust to the measures, and one appointment committee researcher said that the measures could be refined over time if needed on the basis of continuous assessment of issues that arise with implementation and adoption.

When anything new is introduced people are uncomfortable with it, and once they tried it a few times, usually then they develop a certain level of comfort with it and it doesn’t seem so unreasonable. With time, people will learn how to adjust to the new assessment measures and things will be fine (016 ID late biomedical).

DORA has to be a working document, if you will, and one that will be changed with time or with implementation itself (05 AC late biomedical).

Participants said that researchers and evaluators will comply if the measures are formally endorsed as the standard by leadership, and several others recommended an official launch accompanied by a multipronged communication strategy to raise awareness about the measures and, specifically, why they are important, plus training for researchers and evaluators on how to report or assess the measures.

It needs to be made explicit by leaders (011 SS late clinical).

I guess it would be nice to officially launch it somehow and probably that would be with a town-hall or something like that (03 AC mid health services and population).

You need the why are we doing this? Why are we shifting our focus? Why are we asking you not to just count citations and look at journal impact factor (07 AC mid biomedical).

We would require a lot of education for our researchers in guiding them on thinking in this way and using these measures and how best to report it. Education will be critical for our evaluators as well to think in this lens (017 BM).

Specific to helping researchers, a few participants recommended informing researchers about the measures early in their career so that they can track them, and to ensure that researchers have administrative support to help prepare reports based on the measures or, alternatively, automate the reporting process so that data required to report the measures can be directly acquired by administrative assistants from researcher CVs. Specific to helping evaluators, a few participants recommended providing guidance on how to assess and interpret the measures. One participant suggested sharing information across research institutes on approaches they are using to adopt and apply the measures in performance evaluation. A few participants also recommended strategies to ensure fair reviews including: evaluators should be from the same discipline as the researcher under review, multiple evaluators should be employed, and evaluators must contextualize measures to both discipline and career stage.

## Discussion

Through interviews with 18 participants representing a range of roles, disciplines, and career stages affiliated with 6 research institutes, we generated important insight needed to inform implementation of the 10 DORA-compliant measures of research quality and impact identified in our prior research [5]. Participants identified numerous strengths of the measures. In contrast to the many participants who viewed the measures as relevant across disciplines, the main limitation articulated by a few administrators was lack of relevance of the measures across disciplines. Few participants identified barriers to reporting the measures. Several participants thought it would be difficult to judge researchers of different disciplines and to translate the measures into an overall evaluation, resulting in greater workload, and contributing to reluctance to change from the status quo. Key strategies viewed as necessary to overcome barriers and support implementation of the measures included high-level endorsement of the measures, an official launch accompanied by a multipronged communication strategy, training for both researchers and evaluators.

In comparison with previous efforts that generated principles of research assessment rather than specific measures [2, 3], or discipline-specific measures [4], in prior research, we identified and prioritized measures of research quality and impact that can be applied across disciplines [5], whereas in this study we explored barriers of adopting those measures. Approaches for performance appraisal of physicians and nurses have been developed, but they focus on practice-based measures rather than academic research [17, 18]. Thus, to our knowledge, no other research has empirically examined the implications of research assessment for individual researchers that did not rely wholly or in part on Journal Impact Factor. Regardless of the absence of such research, the research assessment landscape is rapidly changing. A recent Nature editorial underscored these changes, noting that, in addition to DORA and the Leiden Manifesto [1, 2], there are several other efforts to advance research assessment [19]. For example, the European Commission established an agreement among a coalition of more than 350 organizations from over 40 European Union countries to reward integrity, teamwork, and a diversity of outputs in addition to other measures of research quality and impact (REF). The Coalition for Advancing Research Assessment Agreement includes principles (e.g., focus research assessment criteria on quality; ensure gender equality, equal opportunities and inclusiveness) and processes to implement these principles (e.g., base research assessment primarily on qualitative evaluation, commit resources to reforming research assessment; raise awareness of research assessment reform). The UK Future Research Assessment Programme (https://www.jisc.ac.uk/future-research-assessment-programme#) will soon issue a report on modernized measures and strategies for research assessment in England, Scotland, Wales, and Northern Ireland. The International Network of Research Management Societies (INORMS), formed in 2001, includes research management societies and associations from across the globe. INORMS generated the SCOPE Framework for Research Evaluation, a five-stage model for responsible assessment: start with what you value, consider context, options for evaluating, probe deeply, and evaluate your evaluations [20]. The Hong Kong Principles refer to five principles of research rigor rather than impact generated through discussion and consensus of over 100 participants at the 6th World Conference on Research Integrity: assess responsible research practices, value complete reporting, reward the practice of open science, acknowledge a broad range of research activities, and recognize essential other tasks like peer review and mentoring [MOHER]. Project TARA (i.e., Tools to Advance Research Assessment) was launched in October 2022 by the DORA initiative to identify, understand, and make visible the criteria and standards universities use to make hiring, promotion, and tenure decisions, and features an online repository of tools (https://sfdora.org/project-tara/). While it is beyond the scope of this study to compare the principles underlying these initiatives, the included principles and processes do appear to support the measures we generated in prior research [5] (e.g., assess research quality, base research assessment primarily on qualitative evaluation), and the recommendations of researchers we interviewed in this study about how to implement the measures.

In this study, while perceived strengths outnumbered limitations, further efforts to promote adoption of the measures must address the limitations identified by participants, warranting some analysis here of each concern. Some participants noted a lack of measures reflecting what is often referred to as service, including activities such as teaching, mentorship, committees, and leadership. We chose to focus only on the assessment of research quality and impact because the emphasis of DORA is on research assessment [1]. However, research institutes or other agencies that evaluate researchers could choose to also assess service. One appointment committee researcher said that a 5-year time frame was too short. We chose this because our organization reviews senior scientists every five years. However, organizations that evaluate researchers could impose a different time frame more suitable to differing evaluation contexts (i.e., hiring, annual salary review, promotion, tenure). One researcher noted that some options for research outputs were not listed as examples on the reporting template. The reporting template was not meant to be exhaustive and specifies that researchers can add outputs of relevance to their research. However, we will add the suggested outputs to the list of the examples to be inclusive of the range of research disciplines. Similarly, one administrator noted the absence of failed attempts at acquiring research funding, a measure that was initially included on our list but did not achieve consensus during the Delphi survey in our previous study [5]. However, we could add failed attempts as an example of response options to the template under the measures of challenges to productivity or other information.

Participants identified several strategies to support adoption of the measures. To help researchers, such assistance could take two forms. A few administrators thought that some researcher may be disadvantaged if they lack ability to describe the value of their research. While researchers are routinely required to write succinct and convincing research funding applications, journal articles, and meeting abstracts, assessing the ability of researchers to describe the merits of their research was beyond the scope of this research. However, to support researchers, we could develop sample reports that demonstrate how to complete the template. To further help researchers, administrative assistants could adapt information contained within academic CVs, which usually include a narrative section on research achievements. To help evaluators, participants recommended guidance on how to translate responses to the ten measures into an overall evaluation. Given that DORA recommends a broad range of measures including qualitative indicators of research impact [1], the measures are not easily quantifiable, which poses challenges for evaluators accustomed to relying on journal metrics. Further research is likely necessary to develop a framework or other guidance to help evaluators determine if a given researcher has been sufficiently productive in a relatively objective rather than subjective fashion. Further, to eliminate bias, participants offered three recommendations to ensure fair reviews: evaluators should be from the same discipline as the researcher under review, multiple evaluators should be employed, and evaluators must contextualize measures to both discipline and career stage. Evaluators likely do consider these factors; however, in our ongoing work to generate a framework for interpreting the measures, we could investigate how to make considering of those factors explicit rather than implicit.

Strengths of this research included use of robust qualitative methods that complied with reporting criteria and standard techniques for ensuring rigor [13–16]. The research was guided at multiple points by input from the interdisciplinary research team. Furthermore, we interviewed participants who varied by role (research institute business managers and directors, researchers and researchers on appointment review committees) and career stage. Participants were affiliated with six research institutes, thus representing the perspectives of a wide range of research disciplines. We do acknowledge some limitations. All participants were employed at a single hospital corporation featuring multiple research institutes. While this may have resulted in similar perspectives or possible bias in favor of the measures, data revealed differing views and recommendations among participants about the merits of the measures. While participants represented all 6 research institutes, given that only 18 researchers participated, they may not have represented all possible views of all healthcare-relevant research disciplines in Canada or elsewhere. Participants included only two of six research institute directors and only three researchers not on appointment review committees, so their views may not be fully represented in the data. We attribute this to timing of recruitment (summer) and burnout among clinician investigators (due to COVID-19). We were not given ethical approval to collect information about sex, gender, or cultural group, so we could not analyze data by these demographic characteristics. While limited sampling may have influenced the results, we achieved informational saturation of themes and identified differing perspectives among the 18 participants. The participants were affiliated with a hospital corporation in Ontario, Canada, so findings may not be transferable or relevant to other locations in Canada or academic institutions elsewhere with differing research assessment rubrics and processes.

## Conclusions

While participants identified many strengths of the ten DORA-compliant measures for research assessment, they also identified some limitations and concerns. At the same time, they offered corresponding strategies to overcome those barriers. Many noted barriers can be relatively easily addressed through high-level official endorsement, communication, training for researchers and evaluators, and modification of the reporting template. Ongoing work is needed to develop a framework that evaluators can use to translate the measures into an overall assessment in response to a key concern about arriving at an overall assessment for an individual researcher. Given little prior research that identified research assessment measures and strategies to support adoption of those measures, this research may be of interest to academic organizations beyond our hospital as well as researchers, funders, and others. Other initiatives (e.g., Hong Kong Principles, Project TARA, CoARA Agreement) can also provide guidance for creating a research culture conductive of DORA-compliant measures.

## Supplementary Information

**Additional file 1.** Prioritized DORA-compliant measures.

**Additional file 2.** Interview guide.

**Additional file 3.** Interview data.

## Data Availability

All data generated or analyzed during this study are included in this published article and its supplementary information files.

## Abbreviation

DORA: San Francisco Declaration on Research Assessment
