# Supplementary material for: Exploring the merits of research performance measures that comply with the San Francisco Declaration on Research Assessment and strategies to overcome barriers of adoption: qualitative interviews with administrators and researchers
Source: Health Res Policy Syst. 2023 Jun 5;21:43. doi: 10.1186/s12961-023-01001-w (PMC10243029; doi:10.1186/s12961-023-01001-w)
Supplement: Supplementary file 1 — Additional file 1. Prioritized DORA-compliant measures. [file 12961_2023_1001_MOESM1_ESM.docx]

**Additional File 1. Prioritized DORA-compliant measures**

The UHN Research DORA Advisory Group employed a rigorous process to generate consensus across UHN Research Institutes on evidence-informed measures that are compliant with principles of the Declaration on Research Assessment (<https://sfdora.org>) by which to assess researchers for the purpose of appointment or promotion. For more information about the work of the DORA Advisory Group, see here for the [full report](https://universityhealthnetwork-my.sharepoint.com/:w:/g/personal/roberthc_chen_uhnresearch_ca/Ee5gpAjOG1VOgNdAZS8vKiABmFxAoCZz-dcjIOVLfrMDfA?e=SkXbuQ) and [policy brief](https://universityhealthnetwork-my.sharepoint.com/:w:/g/personal/roberthc_chen_uhnresearch_ca/EUU3M3BfpHBIpziEmU4K82AB3CXlGbWUJCatQnGn670F5g?e=Y4J4ZV). If links don’t work, please try:

Full report: <https://universityhealthnetwork-my.sharepoint.com/:w:/g/personal/roberthc_chen_uhnresearch_ca/Ee5gpAjOG1VOgNdAZS8vKiABmFxAoCZz-dcjIOVLfrMDfA?e=SkXbuQ>

Policy brief: <https://universityhealthnetwork-my.sharepoint.com/:w:/g/personal/roberthc_chen_uhnresearch_ca/EUU3M3BfpHBIpziEmU4K82AB3CXlGbWUJCatQnGn670F5g?e=Y4J4ZV>

**Relevance of Research Program**

1. **Describe how your research advances existing applied knowledge, theoretical knowledge or both (5 bullet points)**

Identify gaps in knowledge that your research seeks to address. Note why it is important to pursue this research, and specify the likely benefits and their application

1. **Describe what is innovative about your research (5 bullet points)**

Describe novel themes, methods, data or other outputs, and how they address the aforementioned gap(s) in knowledge

1. **Describe how your research directly or indirectly contributes to the health and health care of Canadians. (3 bullet points)**

For those doing basic science research, note the potential applications of your research that may ultimately lead to innovations or interventions that improve health or health care

**Research Outputs**

1. **List and describe up to 5 peer-reviewed publications from the last 5 years that best illustrate the contributions of your research to the field.**

Describe what is novel about the research approach, note key findings, specify why/how findings are unique and important, highlight implications for ongoing research, or science, practice, policy or society. Do NOT report journal-based metrics such as Journal Impact Factor.

1. **Describe key research outputs other than publications (5 bullet points)**

Choose those relevant to your discipline not featured in publications from the following list or provide other relevant options:

- Commercialization of technology (e.g. software, drugs, devices), launch of companies, invention disclosures, patent applications, issued patents
- Creation of cohorts or registries
- Reusable software or datasets
- Reusable reagents (plasmids, mouse models, cell lines)
- Clinical tests, algorithms or statistical models
- Validated questionnaires or instruments
- Contribution to policies, standards, guidelines or programs
- Novel theory, model or framework
- Novel research approaches or methods
- Other forms of achievement or outputs relevant to discipline (e.g. performance art)

1. **Highlight recognition or dissemination of research outputs other than publications (3 bullet points)**

For example: Describe participation in meetings, workshops or other events as an invited speaker, awards or recognition by academic or professional groups, and targeted dissemination other than publications (e.g. social media). Do NOT report h-index or similar metrics.

**Funding**

1. **Describe currently-held peer-reviewed research funding**

Itemize distinct research projects, noting support from peer-reviewed research funding. For Role, specify PI, co-PI or specific key role such as biostatistician, qualitative expert. Add rows as needed.

| Project title | Funder | Years | Total Amount | Role |
| --- | --- | --- | --- | --- |
|  |  |  |  |  |
|  |  |  |  |  |
|  |  |  |  |  |
|  |  |  |  |  |
|  |  |  |  |  |

**Collaboration**

1. **Provide evidence of local, regional, national or international scientific leadership or collaboration (3 bullet points)**

Describe examples of local, regional, national or international team science contributions (e.g. formation of clinical network, member or leader of an inter-/multi-disciplinary research group)

**Challenges to Research Productivity**

1. **Describe challenges faced in the last 5 years (5 bullet points)**

This includes challenges to research; leaves of absence due to health, family or other circumstances. Note how those challenges affected your research and any mitigating strategies applied to manage or overcome those challenges.

**Other Information**

1. **Further information about the quality and impact of your research not described above (5 bullet points)**

For example: impact of your research relevant to research discipline and career stage, non-peer reviewed funding awarded, attempts to capture funding, engagement of stakeholders in co-production of research
