# Supplementary material for: Exploring the merits of research performance measures that comply with the San Francisco Declaration on Research Assessment and strategies to overcome barriers of adoption: qualitative interviews with administrators and researchers
Source: Health Res Policy Syst. 2023 Jun 5;21:43. doi: 10.1186/s12961-023-01001-w (PMC10243029; doi:10.1186/s12961-023-01001-w)
Supplement: Supplementary file 2 — Additional file 2. Interview guide. [file 12961_2023_1001_MOESM2_ESM.docx]

**Additional File 2. Interview guide**

- Thank you for speaking with me today. To briefly review the purpose of this interview…
- UHN endorsed DORA, a set of principles that discourage metrics like journal impact factor and encourage measures of research quality and impact to assess researchers for hiring and promotion
- The DORA Advisory Group compiled measures from published research and those used by national and international academic organizations, and surveyed UHN researchers to establish 10 priority measures of research quality and impact – we sent the measures to you by email – hopefully you had a chance to look at them and have them in front of you today.
- We will discuss use of those measures. The findings will help us to refine the measures and identify strategies needed to suppose use of the measures.
- We are audio-recording the interview to create a transcript for analysis. Please remember to speak slowly and loudly so that the audio-recording is clear <***make sure you can hear them***>
- Before we begin, do you have any questions?

MERITS [First let’s discuss the merits of the measures….]

| Question | Prompts |
| --- | --- |
| Please describe what you perceive as the strengths of the measures | - Rigorously developed (based on evidence, practices used elsewhere and consensus at UHN) - Address all the major aspects of being a productive researcher - Relevant to your research discipline or research institute - Not too different from current measures - Standardize reporting/assessment across UHN research institutes - More equitably assess researchers across disciplines |

LIMITATIONS [Next, let’s discuss any problems you see with the measures…]

| Question | Prompts |
| --- | --- |
| Please describe what you perceive as limitations or gaps in the measures | - Not relevant or equitable for all research disciplines - What measures would be more relevant for your research/research institute? - What measures do you think are missing? |

BARRIERS TO REPORTING [My next question is about barriers of reporting your research with these measures…]

| Question | Prompts |
| --- | --- |
| What do you think might be barriers of using these measures to report research? | - Difficult to track or describe - Why? Please elaborate |

BARRIERS [Now I will ask about barriers of using the measures to assess researchers…]

| Question | Prompts |
| --- | --- |
| What do you think might be barriers of assessing researchers based on these measures? | - *Even if not an appointment committee member*: What concerns do you have about someone evaluating you based on these measures? - Measures are qualitative so evaluation is subjective - Requires considerable expertise in the same research discipline - Focused only on research; what about teaching, supervision, etc. - Process/logistics – do researchers still submit a CV? - Weighting of measures; which ones are more important? |

STRATEGIES [I have one more question…]

| Question | Prompts |
| --- | --- |
| What strategies are needed to raise awareness and facilitate use of the measures for reporting or assessing research? | - Use different measures or add measures - Email reminders - Training for researchers or appointment committee members - Guide/instructions on how to report or assess measures - Adoption/endorsement from the head of the research institute - Champions among appointment committee members in each research institute - Endorsement from the VP of Research (Brad Wouters) |

Is there anything else you would like to add?

Many thanks for speaking with me today
