# Supplementary material for: Exploring the merits of research performance measures that comply with the San Francisco Declaration on Research Assessment and strategies to overcome barriers of adoption: qualitative interviews with administrators and researchers
Source: Health Res Policy Syst. 2023 Jun 5;21:43. doi: 10.1186/s12961-023-01001-w (PMC10243029; doi:10.1186/s12961-023-01001-w)
Supplement: Supplementary file 3 — Additional file 3. Interview data. [file 12961_2023_1001_MOESM3_ESM.docx]

**Additional File 3. Interview data**

**Perceived strengths of the measures**

| Theme | Research Institute Leaders and Administrators | Researchers: Scientists and Appointment Committee Members |
| --- | --- | --- |
| Measures cover the major aspects of being a productive researcher / comprehensive | I think its looking at the big picture. It kind of reaches all areas of things we would essentially evaluate individuals on; like for as an example, in our statements right now its we’re expecting people to develop a world-class research program with potential; with a potential or major impact in the field. And this kind of, is like the steps or ways to actually show that impact and that’s what I liked about that. (04 BM)  This adopts a more holistic approach when we are talking about reviewing people. We want to know if you’ve done something innovative, if you had some impact, if your research has resulted in best practice guidelines being shared with the community; that kind of thing (08 BM)  it looks pretty comprehensive. There is an opportunity to highlight things which are really, we talk when we review or assess research we look at three major things normally; grant funding, publication and awards and those kinds of things… it covers everything (010 BM)  These measures will enable us to evaluate quality and excellence and impact, which is important. (016 ID late biomedical) | I think they address very well what we want to achieve and want our researchers to do. I think they are measuring what we expect…The research outputs are very much the recognized output of academic and translational medial research (01 AC late Biomedical)  It’s more comprehensive for sure because right now we really use only publication metrics and grants. (03 AC mid health services and population)  Well I think they’re comprehensive, fair. (06 AC mid health services)  I thought it was pretty comprehensive actually. (011 SS late clinical)  I think they all seem to be reasonable to me. They seem like they’re good measures of productivity. The ten questions are pretty diverse and you can get a good feel for what that researcher is doing as well as their productivity or of any challenges they have. I’m able to highlight my work through these ten questions. Using these ten criteria as an assessor, I think I can get a pretty good feel for what that researcher is doing and what their successes have been and what their challenges have been (012 CS) |
| Measures are relevant and applicable to different research disciplines | All of them are relevant to be honest. (04 BM)  Yes I think so because it allows overall; overall more inclusive reviews of different areas of the scientist and that will not be limited to as I mentioned earlier before; not limited to just publication. It would encourage reviewers to look at other metrics such as their contribution in for instance, mentorship, supervising students and then there are other areas such as; their commercialization patents, licensing as well as what they have done in terms of tools that they have developed or even what they have done on the social media to, to be done as part of knowledge translation. (015 BM)  I think one of the strengths is the breadth of and the various different angles that are being used to assess the strengths of a research program that takes into consideration or acknowledges the fact that there is a wide range of research and research approaches being conducted whether it is at UHN or at another organization. (017 BM) | How did your research advance the objectives of the research institute for example, because people will be in different departments and institutes, right, with slightly different goals. It’s definitely appropriate for the basic and translational. For the vast majority, I think almost everybody that we review, it really covers well (01 AC late biomedical)  Yah, I think they’re relevant (03 AC mid health services and population)  Again, for my discipline and for the kinds of people I work with in my institute and other partner institutes I think that this is completely appropriate. As a researcher that mostly does basic science, I think this is well designed. (014 AC late biomedical)  There is diversity and variability which then allows for the relevant factors that might be discipline-specific to be taken into account. (011 SS late clinical)  I’m not a basic scientist. I feel like it does cover all disciplines. I feel like it would apply to the different disciplines. (012 CS) |
| Moving away from impact factor allows researchers to personalize reporting, resulting in fair assessment | It goes away from the journal impact factor and basically it enables the researchers to in their own words indicate the relevance of their research program, the strengths of their research outputs and the support for their research programs. (016 ID late biomedical)  Will allow reviewers to review scientist appointments at the overall package but not nail down or in particular focusing only or solely on their publication. (015 BM)  Traditional measures may not look at things such as type of outputs such as commercialization or influence to policy things of that nature and so or even advancing knowledge which sometimes could be harder to quantify. I think that they DORA measures are attempting to break away from strictly quantification of the use of quantification in evaluating the strengths of a research program (017 BM)  What their research is trying to address as opposed to just looking for the next publication and the higher impact factor for that publication would mean a better ranking or a better score for the investigators. I think it helps them to really think about what their research; what their research is articulated and also to have a different focus. Not just thinking about what the impact factors gonna be and where they’re gonna publish but also about what the impact is gonna be and what their trying to accomplish from their research. (018 BM) | What I perceive as the strengths of the measures is the fair assessment of research advances and of individuals involved in research. The measures are something that is going to help us to have a far more fair assessment of what researchers means to the institutions that they’re in. DORA is a welcome addition to our efforts to evaluate research progress in individuals conducting research. We live in times when journal impact factor has become the norm in evaluating people, and journal impact factor is not driven necessarily by the quality of the conducted research but by marketing and PR efforts of the journals themselves because scientific publishing is a highly profitable business (05 AC late biomedical)  I think they are more relevant to what scholarship is and what’s called the activity is and just publications. (06 AC mid biomedical)  Instead of relying on metrics such as; impact factors or journals in which they’re publishing it sounds like this would offer an opportunity for some narrative where they can create those connections and describe that impact in more sort of nuanced and tangible ways rather than just counting, which sounds like a good idea. (07 AC mid biomedical)  I thought that the questions about innovation and outputs kind of captured as well as the recognition and dissemination captured the metrics that are perhaps less traditional such as number of publications and impact factors. (011 SS late clinical)  I mean I suppose it’s that they are less based on numeric measures that have minimal basis behind their use in evaluating quality. (013 S early biomedical)  I’m completely supportive of what I understand of the DORA initiative and trying to modernize how we do our assessments getting away from journal impact factors. Some of the journals game the system, so artificially inflate their impact factor, so it’s a far from perfect measure of research impact. (014 AC late biomedical) |
| Measures are similar to those in current use | I think its great. I mean, a lot of them we’re already using. (04 BM) | They’re very much in-line with what we do now but it’s just a little more laid out…from my point of view, these are all the same measures that we at <institute> use for our evaluations. It’s just structured in a nice way (01 AC late biomedical) |
| Easy to understand and use; response options are limited to key achievements, which minimizes reporting burden, and template provides examples | I know in our evaluations right now a lot of it is just publications but it doesn’t also suggest certain examples which I think I really appreciated (04 BM) | This document is short, succinct and very easy to understand (01 AC late biomedical)  I think this document is great for everybody to see and it’s not very long, so I like that (01 AC late biomedical)  I can see like the relevance of the research program is pretty clear. The research output are pretty clear. (02 AC early health services)  This document is short, succinct and very easy to understand (01 AC late biomedical)  I think this document is great for everybody to see and it’s not very long, so I like that (01 AC late biomedical)  I can see like the relevance of the research program is pretty clear. The research output are pretty clear. (02 AC early health services)  What I do like is that its restricted to say, 5-bullet points or 3-bullet points. So that, there …really long list and it’s forcing people to pick other ones that are most important. (03 AC mid health services and population)  Reasonably concise everybody that’s filling out this kind of forms is busy and were also doing similar things for <research institute> and reporting for funding agencies and that kind of thing. Its nice to have something that’s a straight forward template. (014 AC late biomedical)  Wouldn’t be difficult for the PI to go through these ten and add their bullet points to describe what they do. It seems to be practical, efficient. (012 CS) |
| Measures were generated using a rigorous scientific process |  | And I was thinking that a lot of it is very science-based and makes sense for UHN. (02 AC early health services) |
| Referring to specific measures | Challenges to productivity:  Next is challenges to research productivity; over here it’s always the question of funding, right? I mean you apply for a grant and then your grant is approved but oh well, they’ve got 10% or 20% or whatever and now you have to scramble to try and get your project within the smaller resources that are now available and of course recently with COVID without people being able to have research …in their study. They’ve had to rely on data, systematic reviews, scoping reviews; that kind of thing for these last two years. So definitely they’ve been impacted by research part, lack of research participants. (08 BM)  Collaboration:  The fourth thing is collaborative work. I don’t think; I haven’t seen the other institutes metrics but I don’t think we explicitly ask them or a description of their collaborative work. We infer this information from these group grants whether or not they’re leaders on these group grants and basically it would be extensive institutions or disciplines that they participate in. We infer their collaborative work from that. I think the question here is a little cleaner in a sense that it gets them to describe it and to explain how they’re advancing knowledge or having a larger impact through their collaborative work. It’s a little bit more explicit rather than an implicit description that was; that’s what; which is we did before. (018 BM)  Research Contributions:  I think it allows the researcher to expand on the impact of their work. (09 ID mid biomedical)  We’re expecting people to develop a world-class research program with potential; with a potential or major impact in the field. And this kind of, is like the steps or ways to actually show that impact and that’s what I liked about that. (04 BM)  The first is question 1; I think help which is to describe how your research advances existing applied knowledge, theoretical knowledge or both. I think this is beneficial compared to what it was before because it helps to get the researchers to articulate what their research impact is. (018 BM) | Challenges to Productivity:  Opportunity for someone who may have extenuating circumstances to explain if there are challenges (01 AC late biomedical)  I also think that there; in particular …where it talks about describing challenges based in the last 5-years. I think is a good way of accounting for things that might have changed, pregnancy or pandemics or changes in position or those kinds of things. (06 AC mid biomedical)  The challenges to research productivity section, was it section 9 I think. That’s an important thing because the real world does impact research productivity and so giving folks a space to explain that and explain that beyond what its called more obvious things like a leave of absence as an explanation for interruption of research productivity. sometimes there’s things that maybe impactful, circumstances that’ll be impact that don’t necessarily rise to taking a leave but you have a chance to document that I think is appropriate. (014 AC late biomedical)  Describe what your research is and some of the challenges that you have in some of the outputs you have. So yah, I think it comes back to practicality. (012 CS)  Collaboration:  I like the fact that collaboration is encouraged here or at least one is given the opportunity to explain how they collaborate (01 AC late biomedical)  I was just gonna say, one of the strengths I think is that it asks about collaboration and describes like the different levels; like local, regional, national, international. (02 AC early health services)  Collaborations, I also think is really important because the extent to which you work broadly with others and you embrace interdisciplinary and inter-professionalism and have some obvious evidence that others value your inclusion in their work through collaboration are all gonna be important; important criteria to evaluate. I think collaborations that have tangible output is also something to consider. So collaborations are great but collaborations that lead to something are even better. (07 AC mid biomedical)  Research Contributions:  Laying out clearly the ways one would measure that output. Perhaps in a more consistent way, not with the impact factors, but with other measures. I like the list of key research outputs. It gives you things like databases and computational and informatics tools and public domain resources that are so important these days. Researchers developing those might not even always be published in a traditional peer-review paper. I really like that you have it explicitly stated those kinds of outputs are something that can be highlighted (01 AC late biomedical)  I really appreciate the other information section. And I appreciated the list of key research outputs other than publication. And I think the question that I thought was most important; it was describe how your research directly or indirectly contributes to the health and healthcare of Canadians because I think that that part allows space for people who are working more towards social justice or systems change in their research to be able to explain why their work matters in ways that’s beyond sort of methodological or other forms of scientific contributions. (02 AC early health services)  We are giving them the opportunity to describe how their research advances existing applied knowledge, theoretical knowledge or both. (07 AC mid biomedical) |

**Perceived limitations or gaps in the measures**

| Theme | Research Institute Leaders and Administrators | Researchers: Scientists and Appointment Committee Members |
| --- | --- | --- |
| Measures do not reflect non-research activities such as teaching or service | Any information about supervision or teaching. (04 BM)  Creative professional activities need to be captured, because in certain cases, they do affect how a Research Institute might grow (010 BM) | I’m on the <national committee>. So this where you bring in your scientific expertise and you contribute to the greater good. It’s all volunteer. But there is no recognition for this type of thing (03 AC mid health services and population)  I don’t see any evaluation components that relate to supervision of graduate students or the success of those graduate students? Do we have like outreach and education in there somewhere? That’s another important priority area. If its not described currently in key research outputs other than publications, that might be something else to consider (07 AC mid biomedical)  There’s a lot more that goes into what we do, and I understand that this is supposed to be focused on research, but many of us are also teachers, many of us are mentors and this sort of moves into the realm of not acknowledging those contributions which are fundamental to the operation of UHN Research that really aren’t captured by this. And I think it takes an awfully narrow scope of what’s involved in our roles here. (013 S early biomedical) |
| Output options listed are not comprehensive: |  | Medical education research  But I’m not sure that there are as many suggestions for qualitative outputs as there might be. I think qualitative work might come under the validated questionnaires or instrument; possibly contribution policies, standards, guidelines and programs. I think adding curriculum onto that would capture the health professions education element more appropriately. So I think it was mostly curriculum then I was thinking that should be added to that list. (02 AC early health services)  Intangible outputs of community-based research collaboration  And sometimes I think if you’re working on policy work then scientific leadership doesn’t fit into that category. For example, we’re gonna be collaborating with indigenous health groups across Northern Ontario and they wouldn’t fit into this collaboration and yet we are going to be engaging in data-sharing and probably co-creation of some kind of a written project; possibly a paper if that’s what suits their community. So I think fitting in some of the indigenous requirements for research, like ownership of data and that their research belongs to the community and goes back to the community and doesn’t necessarily turn into a publication; perhaps could be an issue. (02 AC early health services) |
| Measures do not include the effort of failed attempts to capture research funding | What I was thinking is there is another cohort to people who do not; who are not as successful. But I think there is some attempt; there should be some attempt to glean some kind of learning from failed attempts as well, especially on large grants, on what did they learn out of the entire process of applying for a big grant and not getting it. Maybe those kinds of things could be collated and made available to other people who are going on to apply for such grants in the future. That was another thing. It’s not to ignore that cohort of people who do not, who are not as successful in getting larger grants. (010 BM) |  |
| Measures may not be relevant across research disciplines | Some of them might not be as relevant depending on what field they’re in or what stage of their career. (04 BM)  Different disciplines have different kinds of measures. For instance, if you’re reviewing somebody who’s in the computer science field, their publications would not be journal articles. They prefer conference papers and proceedings and that kind of thing. You definitely have to be aware of the discipline that you are reviewing the person you are reviewing and in that context then you would apply the measures. (08 BM)  We have people in social sciences. We have people in library sciences, in bio-ethics and it’s a varied bunch of people. It’s very difficult because what is relevant to one field is not the same as another field. Even without DORA measures it is an obstacle, so with DORA measures even more so (010 BM)  If we used the exact same ten measures for everybody, there’s a possibility that some researchers or the research program will be at a disadvantage because they don’t touch every single point. (017 BM)  Additional parameters that weren’t previously reported, they would now need to keep track of; for instance, creating cohorts and registries, and coming up with new methods and giving cell lines to other labs aren’t typically things that people tracked. From an organizational point of view, it’s important to know that, and from their point of view for their reporting, it’s important to report, but I don’t think they keep track of it. I think it is going to be a little bit challenging to do that (018 BM) | I think the disciplines are so different that to use this collection of measures equally across disciplines is extremely difficult (06 AC mid biomedical) |
| Five-year time frame is too short and may not reflect impact and quality | Some years you could do very well and some years it just doesn’t happen. So again, you shouldn’t be looking at a scientist over just a very small period of time. You need to give them enough put the up’s and down’s of an academic career. (08 BM)  Research outputs again, you can’t just judge a scientist on a particular year because one year they maybe very productive, another year it’s just doesn’t happen. It has to be over a few years that, you track their productivity. (08 BM)  I think one of the deficiencies as I see the program is that it focuses very much on the last 5 years and sometimes I think its important to put things into context over a longer research career; not everything can be accomplished very quickly. Some research projects take over a decade to accomplish and the rationale and other; and other work could be viewed as a; as a more long term goal…I mean some researchers may not have even 5 publications in 5 years. I mean especially if these are; especially if these are substantial publications. I actually think that, that I; when I usually look at a CV I look at 5 most impactful publications over a person’s career to judge them; not, not in the last 5 years. If there is one significant thing over 5 years to me that would be that sort of builds quality over quantity. (016 ID late biomedical) | I don’t t think it sort of gives a, list and describe up to 5 peer-reviewed publications from the last 5 years. I think that implies that if you don’t have 5 then you’ve under performed and I think that for some people and depending on the field that you’re in, 5 is sort of an arbitrary number that really for an early career researcher having 5 peer-reviewed publications in that time period is probably unreasonable. And depending on the field that one is in having one publication of really high quality in 5 years is just as valuable perhaps as 5 not so important ones. (013 S early biomedical)  Well I think it’s sometimes very difficult to evaluate the impact and quality of a particular body of work immediately. (013 S early biomedical) |

**Potential barriers to reporting the measures (researchers)**

| Theme | Research Institute Leaders and Administrators | Researchers: Scientists and Appointment Committee Members |
| --- | --- | --- |
| Time consuming or requires effort | If we know somebody is doing extremely well, is a superstar, do they have to necessarily go through this process? I don’t know if this would be more work, but I feel as if that could be potentially a barrier there. (04 BM)  It’s gonna be a lot of work for people to do this on an annual basis, because as I understand it, the administrative staff goes in, copy from their CV which is a dynamic document that just keeps getting updated as new publications come along. When its time for the annual activity report, the administrative staff assist with pulling these publications into the annual activity report or it’s automatically pulled up by the research analytics group. So it’s basically very simple to complete some sections of the annual activity report. This one’s gonna take a little bit more work to describe how their research is leading to a certain outcome. So it would take that extra thought to put this information together (018 BM) | Right now we assess funding basically from the researchers CV. This is laid out a little bit more rigorous, in a table that’s more structured than different CV’s. The only thing I would say that is a little bit of a negative for the person putting together their report; is that if you have a lot of grants, that could take some time to put together the table and put in the explanation. This is gonna be a little bit more work than just handing in the CV, especially on the output. It puts the burden on the person being evaluated to explain why the research is important and impactful. It’s gonna take time for a researcher to do a good job at communicating the impact and importance of their research, to carefully craft those bullet points to get the point across without writing pages and pages (01 AC late biomedical)  This is an administrative burden on top of many other administrative burdens. And many of us have a number of different places to be accountable to and this is just one more thing to do. It could potentially be a big administrative burden (06 AC mid biomedical)  These things take time to do. Is the PI gonna do it? Or is there a way to use your CV more efficiently to fill in these things? Even though I feel like it’s fairly practical and to the point and you still have to sit down and do it (012 CS) |
| Some people are better at describing their research than others but this does not reflect the quality and impact of their research | Some people are really good at showcasing themselves. Some people might not be as articulate. Some people might be better at that than others. Some people not being able to complete the form as good as others (04 BM)  People will differ in their ability to describe the impact of their work but that does not necessarily mean that that’s a reflection of the impact of the work rather, it reflects people’s abilities to describe it. (09 ID mid biomedical)  I think one; I mean related to the review I think one additional challenge would be that the PI’s that are better able to articulate their value, so the ones that are able to demonstrate a really well; will end up with a more favourable rating compared to a person. (018 BM) | Its not as standardized as the publication record (03 AC mid health services and population)  What becomes difficult is that there’s no template. It maybe difficult for people to understand what’s actually being asked and why, and people will answer it very differently. (07 AC mid biomedical)  I wondered why it was chosen to do things as bullet points. Not that its necessarily wrong, but sometimes actually having free-form prose with full sentences gives you a little bit more ability to explain things or provide context. (014 AC late biomedical) |

**Potential barriers to assessing the measures (evaluators)**

| Theme | Research Institute Leaders and Administrators | Researchers: Scientists and Appointment Committee Members |
| --- | --- | --- |
| Inertia or reluctance to implement or adopt the measures | We all know what the top journals are right now. So even if we take away journal impact factors, I think that in the back of everyone’s mind, the reviewer might still be thinking about them (04 BM)  Acceptance of the measures, because any kind of change is difficult. So this is very new way of assessing research and I think it requires a lot of shift in that we deeply hold on to what we’ve been doing, the norms of assessment, and I think the shift to these kinds of measures is the biggest challenge instead of the measures themselves or gaps in the measures (010 BM)  A lot of researchers are very accustomed, especially the ones who are have been with the organization for a long time, to this many publications, this impact factor, this citation number, numbers that they can actually pull from a report. So when you want to start talking about some of the other more qualitative impact, it might be hard to understand (017 BM)  Things are changing in, in science and its not clear to me or in research and its not clear to me as things are changing; how its not clear to me how important some of these are as compared to established measures that have been used in the past. 016 ID late biomedical) | The actual reality is that the majority of the institutions have signed onto DORA but actually continue with practices that do not implement the measures. It has been almost too easy to project that you agree with the DORA measurement by signing onto the declaration but then carrying on without implementing it. That’s by far the biggest problem with this at the moment (05 AC late biomedical)  The problem is they don’t exactly align with how institutions are tracking performance. And so, you end up picking of some things and not others. (06 AC mid biomedical)  It’s not uncommon for people in leadership here to effectively ignore this document and the implementation of these measures. I honestly don’t really think that leadership and those evaluating performance actually really take these that seriously (013 S early biomedical)  When it comes down to having to make decisions for example, around promotion or hiring or review the comparison; the capability to compare across these responses and measures and criteria maybe become difficult; more difficult and that’s not to say that difficult is bad but it may become a barrier if it leads to sort of disruption to the way that things are often done. (07 AC mid biomedical)  I think a primary barrier for the vast majority of the bench lab researchers is that they deeply believe in the merit-based model of research evaluation and they think anything else would be unfair. So I think that’s gonna be the barrier. (02 AC early health services)  The most dominant impact factor publications is the most dominant metric or numeric metric that we have and translate it into H-index, translate it into whatever these things are number of citations and all this other stuff. Those metrics are definitely applied in short listings from a very large pool of candidates for any position or promotion to the next level. So I think that this is by far the biggest challenge in fully implementing this. (05 AC late biomedical)  I would see all as though I need to achieve those “key research outputs” other than publications. When maybe my employer doesn’t care about these other things and what they care about mostly are publications. So that will be one of the issues to overcome, is this disconnect between what this is expecting and what academic institutions are using. (06 AC mid biomedical) |
| Increased workload to assess merits of publications |  | From the evaluator’s point of view, there’s a lot more burden on having to understand it. There’s an expectation that evaluators will actually read the paper and make an assessment of the value of that research from the research article itself, not just from the title of the journal is published in…on my committee, we would expect the main reviewer to look at the papers…Instead of just skimming over a CV, one has to read a research paper and sort of get a feel for it. On the other hand, if the researcher does a good job at explaining why that work is important and impactful, then it should help (01 AC late biomedical) |
| Unclear how to weight different measures to generate or distinguish an overall evaluation | Well there’s no listing of publications among other things. The journal where they’re published although you may argue is not important. I do think in some degree is important and I think those things should be included. I think it lacks specific metrics; data driven metrics about impact. I mean for a publication its pretty clear but I think outside of publications it’s; it becomes a little bit more difficult to, to understand them (09 ID mid biomedical)  How are weighting these measures? And how look at all these impacts and everything but how; how much impact is a big enough impact? Essentially the weighting and I mean the weighting of the criteria or the measures, I guess. Say if somebody is doing fantastic in one area, how and can it off-set how somebody’s doing in another area? (04 BM)  As I said because everyone has different expectation and because the reviewers they are educational; educated professionals in the field. So it’s not going to be easy to, to distinguish between A+, A and A- as I mentioned before. We know that it’s excellent work but how do you define the excellent without a numerical value. (015 BM) | What’s not shown here, and maybe that’s up to the institute, but the weight one would give to each element. I mean, for sure everybody’s research has to be relevant, so that’s yes or no. Then the research output and funding are really the main things that we would evaluate at the end. Collaboration and challenges and other information would probably be modifiers of the output (01 AC late biomedical)  In our system, we have a certain comfort level with the numeric measures. DORA is all about descriptive measures which requires that people read them, understand them and be able to convert them to some sort of an evaluation. And that is in my mind the biggest challenge of DORA. (05 AC late biomedical)  It creates potentially a more complex evaluation of the evaluation criteria if I may if that makes sense. In other words, we maybe able to make a decision based on DORA principles but how do we relate that decision to somebody else’s decision using the same principles. And how does one discuss these measures and cross reference them to each other. How do they interpret those measures across the board in order to come up with a single criteria. That’s what I find to be the most; the most difficult component of this. (05 AC late biomedical)  I suppose the limitation or the barrier here might be working out some kind of a review system that is equitable to take into account all of the varied and diverse metric that might, might be implemented or employed…You can’t just count the number of publications and so on. You have to take into account whether sitting on an international panel of experts to determine a set of guidelines or core outcome measures or something is equivalent to one publication or two publications and so on. (011 SS late clinical)  The challenging part is folks that are in that grey area, that maybe the message needs to be your outputs are not quite there but we think you can; can get there…And whether this would capture; whether this document would give you the information to make that kind of decision. (014 AC late biomedical) |
| Without impact factor, may be difficult to assess research in disciplines that are new or unfamiliar, leading to biased or inaccurate assessments | Unless you are an expert in certain areas you may not, you may not know the quality of the research unless you’re really in that area…Be able to properly assess without being biased and sometimes reviewers evaluate harder on what they are experts in. Whereas, if they’re kind of familiar, they may be a little more gentle just because they don’t know (04 BM)  Assessments become subjective and people’s opinions about the researcher are the personal opinions can cloud the assessment. (09 ID mid biomedical)  If that’s not something that he or she is familiar with, then I’d say it’s hard for that reviewer to comment on or it may be completely left out as part of the evaluation process. (015 BM)  I think things like presentations at meetings in many cases if the meetings are not familiar to the reviewers its difficult to judge how important or impactful that this is in the particular field. (016 ID late biomedical)  Using qualitative approaches can sometimes be subjective…One of the reasons why a lot of people have enjoyed using the traditional measures, which are quite quantitative, is that there’s a perceived easy translation of a 1 to 10 scale where 10 is fantastic and 1 is not, and it’s easy for them to say, well I think you’re an 8, so that’s about an 80%. In qualitative assessment, it could be very subjective at the interpretation of the evaluator and especially when an evaluator is not a content expert…When you don’t have numbers to go by, I’m not sure what that anchor is to help guide our evaluators in providing that assessment…How can evaluators who are not within that field or not doing that type of research effectively evaluate? We are not always able to find an evaluator who is a content expert or who does research in that field to do a particular evaluation (017 BM)  I think the challenge will be getting that metric of their productivity. The annual activity reports contribute to the final scoring for the individual and their performance and their salary increases. So I think there may be some bias that can be introduced if it’s not a person that is very familiar with other research that’s happening in the field and the researcher’s contribution to that specific field. So I think not having the number introduces bias. (018 BM)  The other thing is, is social media. I mean I think that many of us are not very comfortable or understand social media and its impact and how to evaluate that. (016 ID late biomedical) | How that’s interpreted, first of all funding is available to different degrees in different disciplines. And so there might be very large pools of money in some places and very, very little in others and likewise, the diversity of grant opportunities and so on. I think if, to assume that this document of these measures could be consistently applied across disciplines I think would probably be too much of a stretch and that context has to be taken into consideration. (06 AC mid biomedical)  What is problematic I think potentially might be again, in the interpretation of the responses and the inconsistency that people might look for from discipline to discipline. That’s the issue, right? Its not that it’s not representative and appropriate for my particular context, it is. It’s just not, it’s the expectations that are; it’s the interpretation of a result and the use of the results that I think might have some difficulty or isn’t adequately described in this document… it would seem unreasonable for someone in bio-medicine for example, to I think anyways, to interpret the work of a social scientist or an education scientist or humanities or so on (06 AC mid biomedical)  I think UHN, UHN Research in general covers a massive, massive scope of scientific enterprise and social science enterprise that I think is very difficult to expect there it to be; a lot of times we are the only ones who are capable of analyzing the relevance and the placement of what we do in our field. And that makes the whole challenge of evaluating people pretty daunting but it’s also I think fundamental to the issue here. (013 S early biomedical)  That could be a challenge for people working in newer or different areas than most of their peers, right, who may not be able to appreciate the novelty, the impact or the quality of that type of research. So those are areas where having a paper in a peer-reviewed journal with a good reputation, especially an area that you’re not an expert in, is a good sign. But I agree with the impact factor thing…I don’t think that should matter. (01 AC late biomedical)  Especially in the section on describing key research outputs other than publication; then that’s where people will put down what outcomes are important to their field, but if it’s someone from outside of their field, how are they gonna know whether that’s a significant contribution or not…And I think that that’s the place where bias could be an issue because if you don’t appreciate the relevance to a small community then; even though it would have big impact within that community…may not assess that research as being quality or relevant or important (02 AC early health services)  I think those who assess this type of output probably need a bit of training and especially to kind of look beyond your own speciality because its so speciality specific that I think I would find it; I already find it hard to assess, say basic scientist or people who do more qualitative where…I’ve learned over the years. At least to the measures that we usually use like publications and grants of what the differences are across the different disciplines. But to having this; the qualitative measures makes that even harder. So to kind of yah, assess across disciplines I think will be tough, probably the major barrier. (03 AC mid health services and population)  I think one of the issues with the way these things are laid out and I’m one of the reasons people rely on impact factor and those kinds of things now is because they at least have the illusion of being objective. But now with this sort of approach in DORA and one of the sort of downside to that is that everything becomes far more subjective and so you open the opportunity for people to be for bias to enter this and that can be bias for individuals, it can be biased for particular research fields; a variety of things. And so moving away from objective measures into something subjective potentially opens a whole other can of worms. (013 S early biomedical) |

**Strategies needed to address barriers and support adoption of the measures**

| Theme | Research Institute Leaders and Administrators | Researchers: Scientists and Appointment Committee Members |
| --- | --- | --- |
| Allow research institutes to decide if to apply select measures | I think if a research institute, something didn’t kind of resonate with them or wasn’t as important; I think perhaps they could choose not to use one of those measures (04 BM)  We have performance metrics that we share with our scientists so they are aware of the measures and I think we will go back to our performance metrics and see how best to include those measures within that. (08 BM)  Further discussion to decide on how research institutes would like to comply with DORA is a very good idea. On-going conversations with the research members in the Executive Councils would be necessary prior to implementation. (09 ID mid biomedical) |  |
| Researchers and evaluators will comply if measures are formally endorsed as the standard and become normalized | Endorsement of this effort needs to come from the top down and down to everyone. There needs to be champions who are pushing forward this vision. (010 BM)  The leaders can start that and have members within the team engage and empower. (015 BM) | The actual idea of DORA is fantastic. The reality of implementation is you need a complete buy-in and require that the measures be in fact implemented. (05 AC late biomedical)  It would have to be adopted by the institutions to which we are accountable to. And maybe even before that, it has to be in how we talk about research at the training level. For those getting into research for the first time, that these kinds of things be discussed, become normalized, become expectation, become what matter, becomes part of the culture. So all of those things I think has to happen for this to thrive. (06 AC mid biomedical)  First, it comes from leadership. Leadership has to implement this over any formalized protocol or strategy that’s currently used for evaluation of scientists for the purposes of awards, for the purposes of annual reviews, for the purposes of promotion. Some harmonization might be required if we want to ensure that, at minimum, these DORA considerations are actually being implemented. Ensuring some sort of enforcement. I know that <research institute> for example, I serve on the evaluation group for grant reviews, and they have chairs who are monitoring all of the discussions about each application. And any time one of the reviewers who’s presenting their assessment of the application starts to use metrics, they are discouraged. For example, if the reviewer says, this person has published in high impact journals, the chair steps in and corrects them and says, just a reminder we don’t want to use journal impact, can you instead comment on any indicators of impact apart from journal impact. (07 AC mid biomedical)  It needs to be made explicit by leaders what the measure are (011 SS late clinical) |
| Hold an official launch and communicate in various ways to raise awareness on why this is important | It’s really important to get out to everybody. Try and communicate throughout the institutes. (04 BM)  It requires a lot of socialization beforehand. A lot of getting people on board and understanding the vision behind this. We do town halls and open forum, that kind of a thing to better socialize it and make it an open discussion and gather people’s comments and their apprehensions and appreciation of the entire process (010 BM)  I mentioned communication. I think that’s key. Use our traditional email with a lot of rationale, your links to documents, but that doesn’t hit everybody because a lot of people are very inundated with a lot of all-user messaging. (017 BM) | I guess it would be nice to officially launch it somehow and probably that would be with a town-hall or something like that. (03 AC mid health services and population)  And so I think reminding people that this is about equitable assessment of research and moving beyond the old paradigms might be helpful after people have had that training. (02 AC early health services)  I think having the focus on equity, diversity and inclusion in terms of setting this kind of…but also especially in assessing this type of information and the differences across different people would be really important. (03 AC mid health services and population)  Engaging the local research community to raise general awareness You need to ensure that you have people on the committees who are also aware of why this is an approach that UHN is taking. So I think you need the why are we doing this? Why are we shifting our focus? Why are we asking you not to just count citations, etc., and look at journal impact factor. Why are we doing that; is going to be important for anybody who serves on selection committees or chairs on selection committees or evaluation committees or awards committees. (07 AC mid biomedical)  I think educating people in what why it’s important to do it and what will come back to the researcher if you do this. So will there be…on the other side, if there is somewhere they’d be struggling with. (012 CS)  I think people will know why it’s happening but just kind of the how it’s being implemented locally would be useful. (014 AC late biomedical) |
| Ensure that researchers have admin support or automate the reporting process |  | I don’t know what else we could do besides having most researchers have access to an assistant who could move information from someone’s CV into this form. That part could be done by an assistant (01 AC late biomedical)  Is there smart text to link our CV’s in some UHN format and then you can link what they’re looking for in the DORA document to your CV to make it even more efficient. (012 CS) |
| Inform researchers about the metrics so that they can track them | Scientists are aware that this is what you’re being measured on and I don’t think they’ll have any difficulty in meeting the measures. (08 BM) | Knowing these in advance, so if I was preparing my career, knowing these in advance, these can all be tracked. (06 AC mid biomedical) |
| Train researchers or appointment committee members on how to report or assess the measures | Researchers (on reporting)  From time to time scientists need reminders. I would say on-going training will be very helpful. (015 BM)  I think we would require a lot of education for our researchers in guiding them on thinking in this way and using these measures and how best to report it… the education piece will be critical education for our evaluators as well as to our researchers who need to think in this lens (017 BM) | Researchers (on reporting)  Provide some training and tools for some of the items that are more difficult to fill in. (03 AC mid health services and population)  Evaluators (on assessment)  I think absolutely they need training…I think we know from other research that people tend to assess people from within their group more favourably than people from without their group. I think anytime that there’s assessment then there always need to be that anti-bias reminder (02 AC early health service)  Have some kind of training and build that expertise to know what’s relevant in a discipline. (03 AC mid health services and population)  Maybe some brief training modules for people who are serving on these various committees could be helpful. (07 AC mid biomedical)  General  I think training or better dissemination of this information is needed (05 AC late biomedical)  There needs to be transparency about what they are and what the expectations are (011 SS late clinical) |
| Provide guidance on how to assess and interpret the measures | It’s really important for the appointments committees to be very, very clear on what we’re doing and the new measures. They need to be very confident in how they’re gonna start doing the reviews (04 BM)  Resource documents that our evaluators can constantly go back to because they’ll forget in a year. They’ll forget when its time in the fall to do their appointment review, right? Our evaluators are always asking our office at the time of doing their annual activity reviews or the appointment reviews, oh what is the criteria again and how do we do this? We also really need guidance on what is considered a good job and not a good job when we no longer have those numbers to attach ourselves to (017 BM)  Getting the institutes involved in that would be really helpful because it’s gonna be a little bit more work for them. But I think if we can demonstrate to them that this would be better for them then I think that would be; would be helpful. For the annual activity report for instance, to have some sort of guidance on how to measure this. (018 BM) | Communicating and reviewing it with the appointments and promotions committee so that they are fully aware of this and how its intended to work. The major thing will be the appointments and promotions committee who are gonna use this (01 AC late biomedical)  It’s gonna be harder than a simple algebra equation which is kind of what you could do with impact factors before right? If you had “x” number of publications at “y” impact factor gives you some value, you need to be above or below that kind of thing. So I think we’ll need guidance as this is implemented and not to replace one algebra thing (014 AC late biomedical)  Share how other institutes are approaching this (014 AC late biomedical) |
| Evaluators should be from same discipline as researcher under review | We have an Appointments Committee which looks at these assessments but we have team leads who would look at the field if its their field. If its not, I think we need to have at least one expert on the team who can do a proper assessment, because as I said, we are completely interdisciplinary (010 BM)  I think that in order for one to truly understand a research program there needs to be some familiarity with the general area of research. So for example, if one has a basic researcher for some of them its very difficult to evaluate a clinical researcher or a health services researcher because they’re just not familiar with that field, they’re not familiar either with the journals or with the impact in that area. So its important to have the evaluation carried out by individuals that are familiar with that area so that they can fairly evaluate it. (016 ID late biomedical) | There should be at least one reviewer who assesses that from within that specialty or area of research. (03 AC mid health services and population) |
| Employ multiple evaluators |  | If you have more than one person in the assessing these indicators, then hopefully you would have convergence at some point on what the outcome is. (011 SS late clinical) |
| Evaluators must contextualize measures to both discipline and career stage | You couldn’t say across the board a metric would be okay. Because someone’s coming from computer science would be at a disadvantage because they may not have five journal articles but they have ten conference proceedings or that kind of thing. You have to put in the context of the person you’re reviewing. (08 BM) | Also funding. In health professions education, people don’t tend to get multi-million dollar grants. They tend to get smaller grants. I guess also because you never have to buy a lot of equipment. So it’s reduced by an equipment amount. But then also it means that when you go to assess the amount of money that a researcher has brought in, then it is less simply because they don’t have those equipment costs and also because the grants tend to be smaller (02 AC early health services)  Different disciplines are at different degrees of development so that’s why having different measures and possibly even different expectations for the kinds of research methodologies, for instance, that might be relevant or important at different stages might be important to take into account. (011 SS late clinical)  They’re going to need context of that discipline absolutely (013 S early biomedical) |
